# Is the frontal lobe involved in conscious perception?

**DOI:** 10.3389/fpsyg.2014.01063

**Published:** 2014-09-19

**Authors:** Shervin Safavi, Vishal Kapoor, Nikos K. Logothetis, Theofanis I. Panagiotaropoulos

**Affiliations:** ^1^Department Physiology of Cognitive Processes, Max Planck Institute for Biological CyberneticsTübingen, Germany; ^2^International Max Planck Research School for Cognitive and Systems Neuroscience, University of TübingenTübingen, Germany; ^3^Department of Imaging Science and Biomedical Engineering, University of ManchesterManchester, UK

**Keywords:** prefrontal cortex, conscious visual perception, frontal lobe, binocular rivalry, perceptual suppression

When studying the neural mechanisms underlying conscious perception we should be careful not to misinterpret evidence, and delineate these mechanisms from activity which could reflect the prerequisites or consequences of conscious experiences (Aru et al., 2012; De Graaf et al., 2012). However, at the same time, we need to be careful not to exclude any relevant evidence about the phenomenon.

Recently, novel paradigms have attempted to dissociate activity related to conscious perception from activity reflecting its prerequisites and consequences. In particular, one of these studies focused on resolving the role of frontal lobe in conscious perception (Frässle et al., 2014). Through a clever experimental design that contrasted blood-oxygen-level-dependent (BOLD) activity elicited during binocular rivalry with and without behavioral reports, Frässle et al. (2014) suggested that frontal lobe, or a large part of it, may not be necessary for conscious perception *per se*. Rather frontal areas are involved in processing the consequences of conscious perception like monitoring the perceptual content in order to elicit the appropriate report of the subjective experience. In particular, Frässle et al. showed that behavioral reports of conscious experiences resulted in increased and more widespread activity of the frontal lobe compared to a condition without behavioral reports, where spontaneous transitions in the content of consciousness were estimated through the objective measures like optokinetic nystagmus (OKN) and pupil dilation. The authors of this study concluded that “*frontal areas are associated with active report and introspection rather than with rivalry per se*.” Therefore, activity in prefrontal regions could be considered as a consequence rather than a direct neural correlate of conscious experience.

However, a previous study (Panagiotaropoulos et al., 2012) that measured directly neural activity in the macaque lateral prefrontal cortex (LPFC) using extracellular electrophysiological recordings could help to narrow down the role of frontal activity in conscious perception and exclude the contribution of cognitive or motor consequences in prefrontal neural activity during visual awareness. Specifically, the activity of feature selective neurons in the macaque LFPC was shown to be modulated in accordance with the content of subjective perception, without any confound from motor action (i.e., behavioral reports). Using binocular flash suppression (BFS), a paradigm of robust, externally induced perceptual suppression and without any requirement of behavioral reports, neurons in the LPFC were found to increase or decrease their discharge activity when their preferred stimulus was perceptually dominant or suppressed, respectively. Therefore, since neuronal discharges in the LPFC follow the content of conscious perception even without any motor action, the conclusion of Frässle et al. (2014) about the role of frontal lobe activity in rivalrous perception needs to be refined. Prefrontal activity can indeed reflect the content of conscious perception under conditions of rivalrous stimulation and this activity should not be necessarily considered as the result of a motor action or self-monitoring required for active report. Moreover, the results obtained by Frässle et al. (2014) do not anatomically preclude the entire prefrontal cortex from having a role in conscious perception. Specifically, the BOLD activity related to rivalry in their experiment is still present in the right inferior frontal lobe and right superior frontal lobe (Zaretskaya and Narinyan, 2014). Further, activation of dorso- LPFC in conscious perception of Mooney images was also reported in a study that explicitly controlled for activity elicited by motor action (Imamoglu et al., 2012).

It is true that the BFS-related prefrontal activity cannot conclude on a mechanistic, causal involvement of prefrontal activity in driving spontaneous transitions in conscious perception. This is because BFS is a paradigm of externally induced perceptual suppression and is therefore not directly informative about the role of recorded activity in spontaneous transitions. Therefore, the possibility remains open that the kind of prefrontal activity observed in the macaque LPFC during BFS is not a causal factor for conscious perception but rather reflects some other aspects of monitoring that are not directly related to motor action. For example, prefrontal activity could just reflect a read-out from other areas like the inferior temporal cortex (Sheinberg and Logothetis, 1997) that also reliably reflects the content of conscious perception. However, if this is the case, it triggers the question why this activity that closely follows the content of subjective perception is observed in the LPFC even in the absence of any behavioral report. Overall, it motivates further investigation to understand whether prefrontal activity has a mechanistic role in conscious perception or it might underlie some monitoring functions that are not necessarily bound to motor action.

Similar to this debate on the role of LPFC in visual awareness, the last decade witnessed disagreement on whether activity in primary visual cortex reflects subjective perception as monitored with electrophysiology and fMRI (Leopold and Logothetis, 1996; Tong, 2003; Maier et al., 2008; Keliris et al., 2010; Leopold, 2012). Measuring both electrophysiological activity and the BOLD signal in the same macaques engaged in an identical task of perceptual suppression finally provided the solution (Maier et al., 2008; Leopold, 2012). Therefore, in order to investigate and resolve the role of PFC in visual perception, one must take a similar approach that utilizes multiple measurement techniques simultaneously or in the same animal along with a careful experimental design. The experimental tasks should not only segregate the effect of various cognitive processes such as attention or introspection in comparison to awareness (Watanabe et al., 2011; Frässle et al., 2014), but also use an objective criterion to decode the content of conscious experience (Frässle et al., 2014), therefore separating perception-related activities from the subsequent behavioral report. Such an approach could therefore robustly delineate the prerequisites and consequences of conscious experience and reveal the true correlates of conscious perception.

Lastly, although such a multimodal approach could provide us substantial insights into the activity underlying the representation of conscious content, whether or not this activity has a causal role in mediating perception remains to be understood. Although a number of studies indeed point to a causal involvement of prefrontal cortex in conscious perception (reviewed in Dehaene and Changeux, 2011), a systematic study which directly interferes with prefrontal activity during a task of subjective perception is currently, to the best of our knowledge, missing. While utilizing objective criteria as indicators of perceptual transitions, systematic perturbation of the PFC (such as cooling, transcranial magnetic stimulation, microstimulation, or optogenetics) and observing concomitant changes in the temporal dynamics of perceptual transitions could reveal its causal contribution. Indeed, patients with frontal lesions are impaired in their ability to switch from one subjective view of an ambiguous figure to the other (for example see Ricci and Blundo, 1990, but also see a different case study from Valle-Inclán and Gallego, 2006).

We would like to conclude that in formulating our conclusions related to prerequisites, consequences and true correlates of conscious experiences, we need to have an *integrative view* on the available evidence. Our investigations and conclusions about the neural correlates of consciousness must not only entail better-designed experiments but also diverse experimental techniques (e.g., BOLD fMRI, electrophysiology) that could measure brain activity on different spatial and temporal scales (Panagiotaropoulos et al., 2014). Such a multi-modal approach holds great promise in refining our current understanding of conscious processing.

## Conflict of interest statement

The authors declare that the research was conducted in the absence of any commercial or financial relationships that could be construed as a potential conflict of interest.
